# Evaluation of Lung Cancer Risk Among Persons Undergoing Screening or Guideline-Concordant Monitoring of Lung Nodules in the Mississippi Delta

**DOI:** 10.1001/jamanetworkopen.2023.0787

**Published:** 2023-02-27

**Authors:** Raymond U. Osarogiagbon, Wei Liao, Nicholas R. Faris, Carrie Fehnel, Jordan Goss, Catherine J. Shepherd, Talat Qureshi, Anberitha T. Matthews, Matthew P. Smeltzer, Paul F. Pinsky

**Affiliations:** 1 Thoracic Oncology Research Group, Multidisciplinary Thoracic Oncology Program, Baptist Cancer Center, Memphis, Tennessee; 2 School of Public Health, University of Memphis, Memphis, Tennessee; 3 Division of Cancer Prevention, National Cancer Institute, Bethesda, Maryland

## Abstract

**Importance:**

Guideline-concordant management of lung nodules promotes early lung cancer diagnosis, but the lung cancer risk profile of persons with incidentally detected lung nodules differs from that of screening-eligible persons.

**Objective:**

To compare lung cancer diagnosis hazard between participants receiving low-dose computed tomography screening (LDCT cohort) and those in a lung nodule program (LNP cohort).

**Design, Setting, and Participants:**

This prospective cohort study included LDCT vs LNP enrollees from January 1, 2015, to December 31, 2021, who were seen in a community health care system. Participants were prospectively identified, data were abstracted from clinical records, and survival was updated at 6-month intervals. The LDCT cohort was stratified by Lung CT Screening Reporting and Data System as having no potentially malignant lesions (Lung-RADS 1-2 cohort) vs those with potentially malignant lesions (Lung-RADS 3-4 cohort), and the LNP cohort was stratified by smoking history into screening-eligible vs screening-ineligible groups. Participants with prior lung cancer, younger than 50 years or older than 80 years, and lacking a baseline Lung-RADS score (LDCT cohort only) were excluded. Participants were followed up to January 1, 2022.

**Main Outcomes and Measures:**

Comparative cumulative rates of lung cancer diagnosis and patient, nodule, and lung cancer characteristics between programs, using LDCT as a reference.

**Results:**

There were 6684 participants in the LDCT cohort (mean [SD] age, 65.05 [6.11] years; 3375 men [50.49%]; 5774 [86.39%] in the Lung-RADS 1-2 and 910 [13.61%] in the Lung-RADS 3-4 cohorts) and 12 645 in the LNP cohort (mean [SD] age, 65.42 [8.33] years; 6856 women [54.22%]; 2497 [19.75%] screening eligible and 10 148 [80.25%] screening ineligible). Black participants constituted 1244 (18.61%) of the LDCT cohort, 492 (19.70%) of the screening-eligible LNP cohort, and 2914 (28.72%) of the screening-ineligible LNP cohort (*P* < .001). The median lesion size was 4 (IQR, 2-6) mm for the LDCT cohort (3 [IQR, 2-4] mm for Lung-RADS 1-2 and 9 [IQR, 6-15] mm for Lung-RADS 3-4 cohorts), 9 (IQR, 6-16) mm for the screening-eligible LNP cohort, and 7 (IQR, 5-11) mm for the screening-ineligible LNP cohort. In the LDCT cohort, lung cancer was diagnosed in 80 participants (1.44%) in the Lung-RADS 1-2 cohort and 162 (17.80%) in the Lung-RADS 3-4 cohort; in the LNP cohort, it was diagnosed in 531 (21.27%) in the screening-eligible cohort and 447 (4.40%) in the screening-ineligible cohort. Compared with Lung-RADS 1-2, the fully adjusted hazard ratios (aHRs) were 16.2 (95% CI, 12.7-20.6) for the screening-eligible cohort and 3.8 (95% CI, 3.0-5.0) for the screening-ineligible cohort; compared with Lung-RADS 3-4, the aHRs were 1.2 (95% CI, 1.0-1.5) and 0.3 (95% CI, 0.2-0.4), respectively. The stage of lung cancer was I to II in 156 of 242 patients (64.46%) in the LDCT cohort, 276 of 531 (52.00%) in the screening-eligible LNP cohort, and 253 of 447 (56.60%) in the screening-ineligible LNP cohort.

**Conclusions and Relevance:**

In this cohort study, the cumulative lung cancer diagnosis hazard of screening-age persons enrolled in the LNP was higher than that in a screening cohort, irrespective of smoking history. The LNP provided access to early detection for a higher proportion of Black persons.

## Key Points

**Question:**

How does the cumulative incidence of lung cancer for persons aged 50 to 80 years in a lung nodule monitoring program (LNP) compare with that in a lung cancer screening (LDCT) cohort?

**Findings:**

In this cohort study of 6684 participants in an LDCT cohort and 12 645 participants in an LNP cohort, those in the LNP cohort had significantly greater incidence of lung cancer diagnosis within 2 years. Those in the LNP cohort who were ineligible for screening (because of smoking history) had greater hazard than those in the LDCT cohort with no potentially malignant lesions, and those in the LNP cohort who were eligible for screening had greater hazard than those in the LDCT cohort with potentially malignant lesions.

**Meaning:**

These findings suggest that irrespective of smoking history, LNP participants of lung cancer screening age had a substantial hazard for lung cancer.

## Teaser

This cohort study compares the cumulative incidence of lung cancer in screening-age populations within a lung nodule monitoring program and a lung cancer screening program in the Mississippi Delta.

## Introduction

Lung cancer mortality is diminishing in the US, along with downward stage shift and improvement in survival across stages.^1,2,3^ The stage redistribution began shortly after low-dose computed tomography screening (LDCT) became a covered health care benefit.^1^ However, implementation barriers hinder widespread adoption, and a geographic disparity has emerged, including a mismatch between state-level density of accredited LDCT facilities and per-capita lung cancer mortality.^4^ Although residents of states and counties in the Mississippi Delta region have some of the worst lung cancer mortality statistics, fewer than 5% of eligible persons in southern US states have been screened.^5,6^ Paradoxically, lung cancer screening threatens to exacerbate preexisting racial and geographic disparities in lung cancer mortality.^7,8,9,10,11,12^

Persons at greatest risk for lung cancer, such as the less well-educated, rural dwellers, and the socioeconomically disadvantaged, are also least likely to access preventive care.^13,14,15,16,17^ Such persons often seek care in settings ill-equipped for preventive care. The use of CT imaging has been steadily rising in North America,^18,19^ along with the frequency of detecting lung nodules.^20^ Guideline-concordant management of such incidentally detected lung nodules provides another route to early detection of lung cancer.^21,22,23,24^ We concurrently implemented LDCT and lung nodule programs (LNP) within a community-based health care system, and a previous report^24^ found a higher frequency of lung cancer diagnosis through the LNP. It is unclear whether the larger numbers of persons diagnosed with lung cancer in the LNP simply reflect the larger numbers undergoing surveillance through this program, or if the cumulative lung cancer diagnosis hazard differs between the types of persons enrolled.

To evaluate this, we compared the cumulative incidence of lung cancer in screening-age populations within both programs. We hypothesized that lesions scored as Lung CT Screening Reporting and Data System (Lung-RADS) 3 (recommended for CT scan follow-up within 6 months) and Lung-RADS 4 (recommended for radiological follow-up within 3 months or invasive diagnostic testing) infer a hazard of lung cancer diagnosis similar to lesions typically identified by radiologists as needing further surveillance or intervention in the LNP setting.^25,26^ We evaluated the relative hazard of lung cancer diagnosis in subsets of LNP participants with different smoking histories. We also evaluated the association between Medicare eligibility age, race, and access to early lung cancer detection through LDCT and LNP.

## Methods

With permission of the Institutional Review Board of the Baptist Memorial Health Care Corporation, including a waiver of the informed consent requirement for this low-risk quality improvement implementation project, we constructed a prospective observational cohort including all persons in the institution’s LDCT program and the LNP.^24^ The Baptist Memorial Health Care Corporation is a not-for-profit community health care system with facilities across Mississippi, eastern Arkansas, and western Tennessee and a service area population spanning 111 counties, including southwestern Kentucky, southeastern Missouri, and northwestern Alabama. This region has some of the highest US per capita lung cancer mortality rates and the lowest participation rates in LDCT screening.^1,4^ This study followed the Strengthening the Reporting of Observational Studies in Epidemiology (STROBE) reporting guideline.

### Data Sets and Data Collection

In prospective fashion, we identified the LDCT cohort using *Common Procedural Terminology* and Healthcare Common Procedure Coding System codes for lung cancer screening (G0297 and 71271, respectively); we identified the LNP cohort from radiological studies in the health care system, irrespective of type or indication. From 2015 onward, we encouraged radiologists to include a standardized statement (eAppendix in Supplement 1) in their reports whenever they identified a potentially malignant lesion, irrespective of the type of radiological study or the indication for the test. Reports including this standardized statement were captured automatically and placed in a queue by the electronic health record system (Epic Systems Corporation). A trained team of navigators used Fleischner Society guidelines to classify participants into risk strata for subsequent review by a multidisciplinary team of general thoracic surgeons, pulmonologists, and radiologists.^22,23,24^

### Database Construction, Auditing, and Management

Trained data managers (C.F., J.G., C.J.S., and T.Q.) abstracted demographic, clinical, care delivery, and outcomes information from the electronic health records of participants in each program into prospective Research Electronic Data Capture databases.^27^ Race and ethnicity were self-identified. All clinical details, including smoking history, risk factor exposures, radiological tests, radiological findings including lesion characteristics, and pathological findings, were directly abstracted from the electronic health record. We performed regular audits and data validation following prespecified standard operating procedures. Care delivery information and information on lung cancer diagnosis were updated in real time, and vital status was updated at 6-month intervals.^24^ Lung cancer diagnosis was current as of January 1, 2022.

### Inclusion Criteria

**Figure 1.**
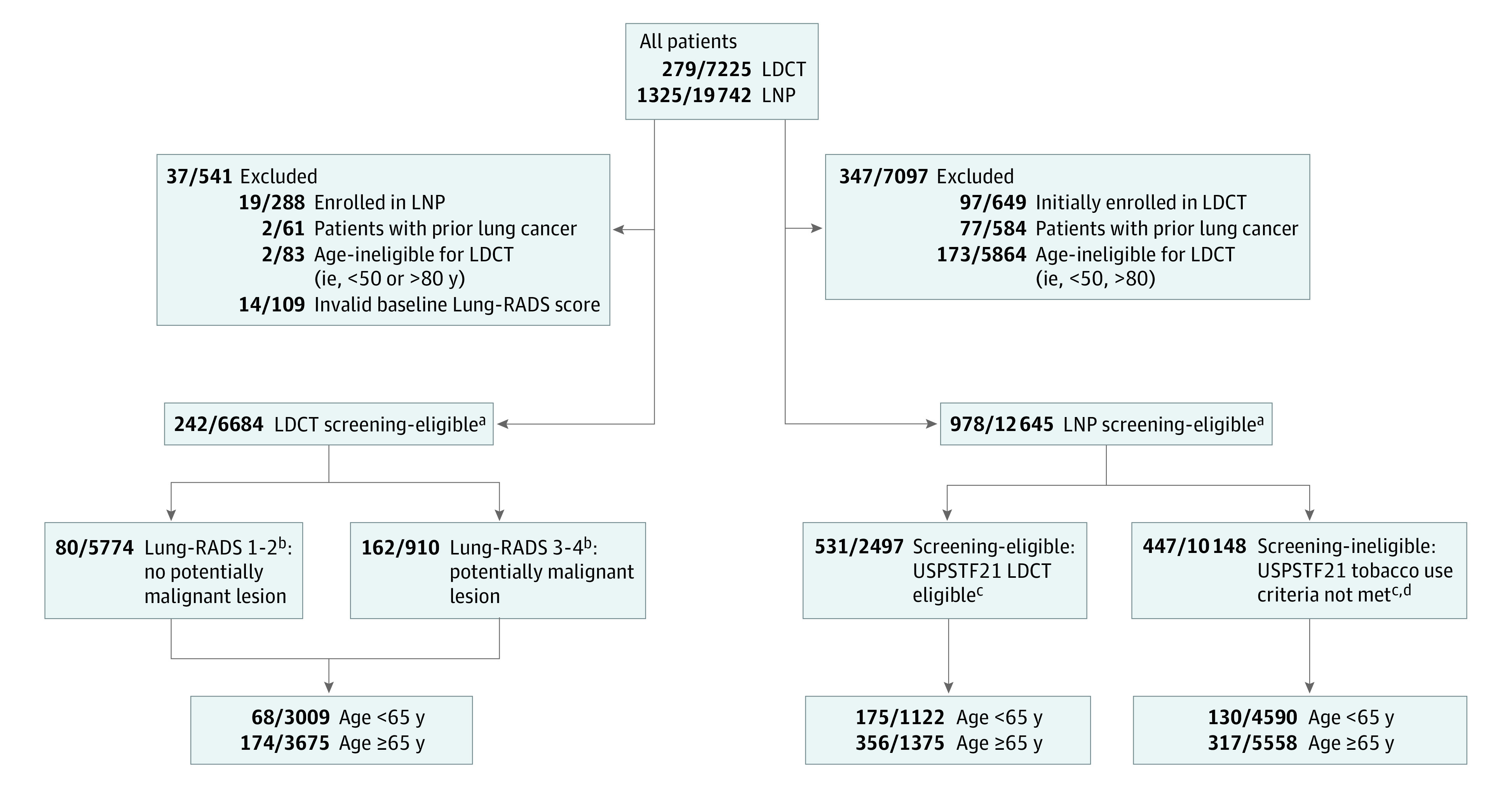
Cohort Selection In all boxes, the numerator indicates the number of patients diagnosed with lung cancer; the denominator, the number of study participants in the category. Patients in the bottom row of boxes were included to examine how Medicare insurance eligibility intersects with access to early detection in US Preventive Services Task Force 2021 (USPSTF21) age-eligible patients in both programs. LDCT indicates low-dose computed tomography (CT) screening program; LNP, lung nodule program; Lung-RADS, Lung CT Screening Reporting and Data System, with scores of 1 (negative), 2 (benign appearance or behavior), 3 (probably benign), and 4 (suspicious or very suspicious). ^a^USPSTF21 lung cancer screening age of eligibility is 50 to 80 years. ^b^Based on baseline (T0) LDCT screening scan. ^c^Screening eligibility in the LNP cohort was also defined according to the USPSTF21 smoking history criteria (minimum 20 pack-years, active smoking, or quit within 15 years). ^d^Participants in the LNP cohort with indeterminate smoking history were also categorized as screening ineligible.

Starting with the full LDCT and LNP cohorts from January 1, 2015, to December 31, 2021, we eliminated persons with a history of lung cancer and those outside the US Preventive Services Task Force (USPSTF) recommended screening eligibility age range of 50 to 80 years, as well as those missing the Lung-RADS score at their baseline (T0) LDCT scan (Figure 1).^28^ For this analysis, participants were assigned to one or the other program based on initial enrollment. We used the LDCT cohort for reference.

### Comparison Groups

We stratified the LDCT cohort into 2 mutually exclusive groups: persons with Lung-RADS 1 (negative) or 2 (benign appearance or behavior) and those with Lung-RADS 3 (probably benign) or 4 (suspicious or very suspicious) scores based on their T0 scan.^25,26^ We also stratified the LNP cohort into 2 mutually exclusive groups: a screening-eligible cohort, meeting USPSTF 2021 smoking criteria (a minimum of 20 pack-years, active smoking, or quit less than 15 years prior to enrollment), and an ineligible cohort that either did not fulfill the criteria or had an indeterminate smoking history (Figure 1).^28^ For additional analyses, we segregated the cohorts into persons younger than 65 years and 65 years or older, the age of Medicare eligibility.

### Statistical Analysis

We used descriptive statistics to summarize the demographic, clinical, and lung cancer characteristics, including frequency (percentage), mean (SD), and median (IQR). For statistical comparisons across groups, we used the χ^2^ test or analysis of variance. We evaluated time to lung cancer diagnosis using time-to-event analyses to account for differential follow-up time and for censored observations. Specifically, we used the cumulative incidence function with competing risks (death) to evaluate the time to lung cancer diagnosis. Follow-up time was measured from T0 (the date of first LDCT screening or initial nodule-detection scan) until the date of diagnosis with lung cancer (event of interest), death (competing risk), last contact or January 1, 2022 (censored). As is common in time-to-event analyses, we assume noninformative censoring. We also reported the total number of lung cancers diagnosed in each group at 6-month intervals.

We fit Cox proportional hazards models and estimated crude and adjusted hazard ratios (HRs and aHRs) for lung cancer diagnosis, interpreted as the mean effects over time. We evaluated the proportional hazards assumption for LNP vs LDCT cohorts using log-log plots (eFigure 1 in Supplement 1). The minimally adjusted models controlled for age at T0 scan, sex, and race and ethnicity as confounders. Fully adjusted models included age, sex, race and ethnicity, chronic obstructive pulmonary disease (COPD) status (yes or no), smoking status, and insurance (unless used as a stratification factor in subanalyses). In additional analyses, we limited the cohort to persons without a history of cancer. We performed complete case analysis when data were missing. All statistical analyses were performed with R, version 4.1.1 (R Program for Statistical Computing). Two-sided *P* < .05 indicated statistical significance.

## Results

### Cohort Characteristics

From 2015 to 2021, there were 7225 persons in the LDCT cohort and 19 742 in the LNP cohort. We restricted enrollees to the program they were first enrolled in, thus excluding 288 persons from the LDCT (prior LNP enrollment) and 649 from the LNP (prior LDCT enrollment) cohorts. We also excluded 61 persons from the LDCT and 584 from the LNP cohorts because of a history of lung cancer; 83 and 5864 age-ineligible persons from the respective cohorts; and 109 LDCT enrollees with a missing T0 Lung-RADS score. The primary analytic cohorts were 6684 enrollees (92.51%) for LDCT (mean [SD] age, 65.05 [6.11] years; 3375 men [50.49%] and 3309 women [49.61%]) and 12 645 (64.05%) for LNP (mean [SD] age, 65.42 [8.33] years; 5588 men [44.19%] and 6856 women [54.22%]) (Figure 1). Black participants constituted 1244 (18.61%) of the LDCT cohort and 3406 (26.94%) of the LNP cohort, including 492 (19.70%) of the screening-eligible and 2914 (28.72%) of the screening-ineligible cohorts (*P* < .001); White participants constituted 5337 (79.85%) of the LDCT cohort and 8638 (68.31%) of the LNP cohort, including 1965 (78.69%) in the screening-eligible and 6673 (65.76%) of the screening-ineligible cohorts. The median follow-up time was 551 (IQR, 235-1025) days for the LDCT cohort and 838 (IQR, 289-1498) days for the LNP cohort.

In the LDCT cohort, 3009 participants (45.02%) were younger than 65 years and 3675 (54.98%) were 65 years or older at the time of their T0 scan. In the LNP cohort, 2497 participants (19.75%) met USPSTF 2021 lung cancer screening criteria, including 1122 (44.93%) younger than 65 years and 1375 (55.07%) 65 years or older; 10 148 (80.25%) were ineligible for screening because of their smoking history, of whom 4590 (45.23%) were younger than 65 years and 5558 (54.77%) were 65 years or older (Figure 1). The demographic characteristics and lung cancer risk factors of persons in the 2 LNP cohorts differed. The screening-eligible LNP cohort was very similar to the LDCT cohort (Table 1 and eTable 1 in Supplement 1). The proportion of uninsured persons was lowest in the LDCT cohort (92 of 6684 [1.38%]) and highest in the screening-ineligible LNP cohort (899 of 10 148 [8.86%]) (*P* < .001).

**Table 1.** Demographic and Clinical Characteristics of LDCT and LNP Cohorts

Characteristic	Cohort^a^		
	LDCT (n = 6684)	LNP (N = 12 645)	
		Screening eligible (n = 2497)	Screening ineligible (n = 10 148)
Age, y			
Mean (SD) [range]	65.05 (6.11) [50-80]	65.24 (7.78) [50-80]	65.46 (8.47) [50-80]
Median (IQR)	65 (60-70)	66 (59-71)	66 (58-73)
Sex			
Men	3375 (50.49)	1257 (50.34)	4331 (42.68)
Women	3309 (49.51)	1239 (49.62)	5617 (55.35)
Missing	0	1 (0.04)	200 (1.97)
Race^b^			
Black	1244 (18.61)	492 (19.70)	2914 (28.72)
White	5337 (79.85)	1965 (78.69)	6673 (65.76)
Other or not reported^c^	103 (1.54)	40 (1.60)	561 (5.53)
Ethnicity			
Hispanic or Latino	28 (0.42)	13 (0.52)	132 (1.30)
Not Hispanic or Latino	6525 (97.62)	2467 (98.80)	9697 (95.56)
Not reported	131 (1.96)	17 (0.68)	319 (3.14)
Insurance			
Medicare	4068 (60.86)	1549 (62.03)	5354 (52.76)
Medicaid	200 (2.99)	88 (3.52)	183 (1.80)
Commercial	2324 (34.77)	746 (29.88)	3712 (36.58)
Uninsured	92 (1.38)	114 (4.57)	899 (8.86)
Smoking status			
Active	4481 (67.04)	1788 (71.61)	1770 (17.44)
Former	2164 (32.38)	709 (28.39)	2983 (29.39)
Never	23 (0.34)	0	4596 (45.29)
Unknown	16 (0.24)	0	799 (7.87)
Pack-years^d^			
0-9	60 (0.90)	0	630 (13.25)
10-19	147 (2.21)	0	581 (12.22)
20-29	262 (3.94)	445 (17.82)	203 (4.27)
30-40	1302 (19.59)	396 (15.86)	165 (3.47)
>40	4178 (62.87)	1656 (66.32)	307 (6.46)
Missing	696 (10.47)	0	2867 (60.32)
Quit duration, years^e^			
0-15	1901 (87.85)	709 (100)	469 (15.72)
16-25	131 (6.05)	0	510 (17.10)
>25	86 (3.97)	0	894 (29.97)
Missing	46 (2.13)	0	1110 (37.21)
Exposure type			
Second-hand smoke	262 (3.92)	151 (6.05)	1702 (16.77)
Asbestos	71 (1.06)	34 (1.36)	61 (0.60)
Radon	1 (0.01)	0	2 (0.02)
Uranium	0	0	0
Comorbidity distribution, No.			
Mean (SD) [range]	1.34 (1.27) [0-9]	1.82 (1.4) [0-8]	1.15 (1.26) [0-8]
Median (IQR)	1 (0-2)	2 (1-3)	1 (0-2)
Chronic obstructive pulmonary disease			
No	3881 (58.06)	1180 (47.26)	8722 (85.95)
Yes	2803 (41.94)	1317 (52.74)	1426 (14.05)
Charlson Comorbidity Score			
0	1916 (28.67)	403 (16.14)	3868 (38.12)
1	3121 (46.69)	1169 (46.82)	3998 (39.40)
2	1647 (24.64)	925 (37.04)	2282 (22.49)
History of cancer			
No	5451 (81.55)	1927 (77.17)	8057 (79.39)
Yes	1233 (18.45)	570 (22.83)	2091 (20.61)
History of lung cancer			
No	1233 (18.45)	570 (22.83)	2091 (20.61)
Yes	0	0	0
Family history of cancer			
No	1725 (25.81)	720 (28.83)	3156 (31.10)
Yes	3272 (48.95)	1341 (53.70)	3736 (36.82)
Unknown	1687 (25.24)	436 (17.46)	3256 (32.09)
Family history of lung cancer			
No	2460 (36.80)	980 (39.25)	2997 (29.53)
Yes	812 (12.15)	361 (14.46)	739 (7.28)

Abbreviations: LDCT, low-dose computed tomography screening program; LNP, lung nodule program.

^a^ Unless otherwise indicated, data are expressed as No. (%) of participants. Percentages have been rounded and may not total 100; not all categories total numbers in column headings.

^b^ Self-reported.

^c^ Includes Asian and Native Hawaiian or other Pacific Islander.

^d^ Includes persons who actively smoke and formerly smoked.

^e^ Includes persons who formerly smoked.

### Smoking History and Other Lung Cancer Risk Factors

In the LDCT cohort, 4481 of 6684 persons (67.04%) actively smoked cigarettes, comparable with 1788 of 2497 (71.61%) in the screening-eligible LNP cohort; by contrast, 1770 of 10 148 (17.44%) in the screening-ineligible LNP cohort actively smoked (Table 1). Among participants who actively smoked or had quit smoking in the LDCT and the eligible LNP cohorts, 4178 of 6645 (62.87%) and 1656 of 2497 (66.32%), respectively, had a known history of greater than 40 pack-years, compared with 307 of 4753 (6.46%) of the screening-ineligible cohort. In the screening-ineligible LNP cohort, 6755 (66.56%) were definitely ineligible because of at least 1 disqualifying tobacco use criterion, including 4596 (45.29%) who had never smoked; 2147 (21.16%) had a history of active or former smoking but were missing all other details with which to determine eligibility, 447 (4.40%) had at least 1 eligibility criterion but were missing other information with which to determine eligibility, and 799 (7.87%) had no information on their smoking status.

Chronic obstructive pulmonary disease was reported in 2803 participants (41.94%) in the LDCT cohort, 1317 (52.74%) in the screening-eligible LNP cohort, and 1426 (14.05%) in the screening-ineligible LNP cohort (*P* < .001) (Table 1). A family history of lung cancer was more common in the LDCT cohort (812 [12.15%]) and the screening-eligible LNP cohort (361 [14.46%]) than in the screening-ineligible LNP cohort (739 [7.28%]) (*P* < .001). Other lung cancer risk factors, such as second-hand smoke, radon gas, and uranium exposure, were infrequently recorded.

### Lesion Characteristics

In the LDCT cohort, 5774 (86.39%) had Lung-RADS 1-2 and 910 (13.61%) had Lung-RADS 3-4 scores; of these, 1032 (17.87%) and 353 (38.79%), respectively, had a single lesion, and 1527 (26.45%) and 517 (56.81%), respectively, had multiple lesions. In the LNP cohort, 1451 (58.11%) of screening-eligible and 5904 (58.18%) of screening-ineligible participants had a single lesion, and 797 (31.92%) and 2877 (28.35%), respectively, had multiple lesions. The number of lesions was unknown in 40 participants (4.40%) in the LDCT Lung-RADS 3-4 cohort, 249 (9.97%) in the screening-eligible LNP cohort, and 1367 (13.47%) in the screening-ineligible LNP cohort (Table 2). The median lesion size was 4 (IQR, 2-6) mm for the LDCT cohort, including 3 (IQR, 2-4) mm for Lung-RADS 1-2 and 9 (IQR, 6-15) mm for Lung-RADS 3-4 cohorts, 9 (IQR, 6-16) mm for the screening-eligible LNP cohort, and 7 (IQR, 5-11) mm for the screening-ineligible LNP cohort.

**Table 2.** Lesion Characteristics of LDCT and LNP Cohorts

Characteristic	Cohort^a^					
	LDCT			LNP		
	Total (N = 6684)	Lung-RADS score		Total (N = 12 645)	Screening eligible (n = 2497)	Screening ineligible (n = 10 148)
		1-2 (n = 5774)	3-4 (n = 910)			
Lung-RADS category^b^						
1	2582 (38.63)	2582 (44.72)	0	NA	NA	NA
2	3192 (47.76)	3192 (55.29)	0	NA	NA	NA
3	455 (6.81)	0	455 (50.00)	NA	NA	NA
4	455 (6.81)	0	455 (50.00)	NA	NA	NA
No. of lesions						
1	1385 (20.72)	1032 (17.87)	353 (38.79)	7355 (58.17)	1451 (58.11)	5904 (58.18)
>1	2044 (30.58)	1527 (26.45)	517 (56.81)	3674 (29.05)	797 (31.92)	2877 (28.35)
0 or not reported	3255 (48.70)	3215 (55.68)	40 (4.40)	1616 (12.78)	249 (9.97)	1367 (13.47)
Size of largest nodule, mm^c^						
Mean (SD)	6.23 (14.22)	3.82 (3.8)	13.36 (26.3)	11 (12.37)	14.52 (16.23)	10.1 (10.99)
Median (IQR)	4 (2-6)	3 (2-4)	9 (6-15)	7 (5-12)	9 (6-16)	7 (5-11)
Missing	72 (2.10)	49 (1.91)	23 (2.64)	829 (7.52)	168 (7.47)	661 (7.53)
Location^c^						
Right upper lobe	1112 (32.43)	856 (33.45)	256 (29.43)	2616 (23.72)	647 (28.78)	1969 (22.42)
Right middle lobe	344 (10.03)	278 (10.86)	66 (7.59)	1281 (11.61)	186 (8.27)	1095 (12.47)
Right lower lobe	624 (18.20)	447 (17.47)	177 (20.34)	2604 (23.61)	443 (19.71)	2161 (24.61)
Left upper lobe	754 (21.99)	569 (22.24)	185 (21.26)	1949 (17.67)	524 (23.31)	1425 (16.23)
Left lower lobe	537 (15.66)	368 (14.38)	169 (19.43)	2101 (19.05)	346 (15.39)	1755 (19.99)
Mediastinum	16 (0.47)	8 (0.31)	8 (0.92)	167 (1.51)	40 (1.78)	127 (1.45)
Not reported	42 (1.22)	33 (1.29)	9 (1.03)	311 (2.82)	62 (2.76)	249 (2.83)

Abbreviations: LDCT, low-dose computed tomography (CT) screening program; LNP, lung nodule program; Lung-RADS, Lung CT Screening Reporting and Data System; NA, not applicable.

^a^ Unless otherwise indicated, data are expressed as No. (%) of participants.

^b^ One indicates negative findings at annual screening; 2, benign findings at annual screening; 3, recommended for CT scan follow-up within 6 months (probably benign); and 4, recommended for radiological follow-up within 3 months or invasive diagnostic testing (suspicious, very suspicious, or additional features or imaging findings that increase suspicion for lung cancer).

^c^ Includes participants with a least 1 reported nodule.

### Cumulative Incidence of Lung Cancer Diagnosis

The median time from enrollment to diagnosis for patients diagnosed with lung cancer was 189 (IQR, 37-658) days for the LDCT cohort, including 779 (IQR, 487-1047) days for the Lung RADS 1-2 cohort and 50 (IQR, 24-225) days for the Lung RADS 3-4 cohort, and 45 (IQR, 14-199) days for the LNP cohort, including 41 (IQR, 12-182) days for the screening-eligible LNP cohort and 51 (IQR, 16-208) days for the screening-ineligible LNP cohort (Table 3). Lung cancer was diagnosed in 242 patients (3.62%) in the LDCT cohort, including 80 of 5574 (1.44%) with Lung-RADS 1-2 and 162 of 910 (17.80%) with Lung-RADS 3-4 scores, and 978 (7.73%) in the LNP cohort, including 531 of 2497 (21.27%) screening-eligible and 447 of 10 148 (4.40%) screening-ineligible persons (Figure 1). Of the 447 participants in the screening-ineligible LNP cohort who were diagnosed with lung cancer, 105 (23.49%) had never smoked cigarettes, 107 (23.94%) had a known history of less than 20 pack-years, and 149 (33.33%) had quit smoking for more than 15 years.

**Table 3.** Cumulative Incidence and Hazard of Lung Cancer Diagnosis

	Cohort					
	LDCT			LNP		
	Total (N = 6684)	Lung-RADS score		Total (N = 12 645)	Screening eligible (n = 2497)	Screening ineligible (n = 10 148)
		1-2 (n = 5774)	3-4 (n = 910)			
Duration of follow-up from enrollment to diagnosis, d						
Mean (SD) [range]	396.53 (451.44) [4-2131]	816.66 (413.66) [35-2131]	191.86 (303.68) [4-1793]	195.88 (338.99) [0-2507]	197.19 (360.49) [0-2507]	194.28 (311.02) [0-1539]
Median (IQR)	189 (37-658)	779 (487-1047)	50 (24-225)	45 (14-199)	41 (12-182)	50.5 (16-208.25)
Cumulative incidence of lung cancer diagnosis (95% CI), mo						
6	1.8 (1.5-2.2)	0.1 (0.02-0.2)	13.0 (11.0-15.0)	5.7 (5.3-6.1)	16.0 (15.0-18.0)	3.1 (2.8-3.5)
12	2.2 (1.9-2.6)	0.1 (0.1-0.3)	15.7 (13.3-18.4)	6.5 (6.1-7.0)	18.5 (16.9-20.1)	3.6 (3.3-4.0)
18	2.9 (2.5-3.4)	0.6 (0.4-0.9)	17 (15.0-20.0)	7.0 (6.6-7.5)	20.0 (19.0-22.0)	3.9 (3.5-4.3)
24^a^	3.4 (2.9-3.9)	1.0 (0.7-1.4)	18.6 (15.8-21.5)	7.5 (7.0-8.0)	21.2 (19.5-22.9)	4.2 (3.8-4.6)
30	4.2 (3.6-4.9)	1.8 (1.3-2.4)	20 (17.0-23.0)	7.8 (7.3-8.3)	22.0 (20.0-24.0)	4.4 (4.0-4.9)
36	4.8 (4.1-5.6)	2.3 (1.7-3.0)	21.0 (17.8-24.4)	8.2 (7.6-8.7)	22.7 (20.9-24.5)	4.7 (4.2-5.1)
Unadjusted model						
HR (95% CI)	1 [Reference]	NA	NA	2.0 (1.8-2.4)	6.3 (5.4-7.32)	1.1 (0.9-1.3)
*P* value^b^	NA	NA	NA	<.001	<.001	.28
HR (95% CI)	NA	1 [Reference]	NA	5.5 (4.3-6.9)	16.7 (13.1-21.3)	2.9 (2.3-3.7)
*P* value^b^	NA	NA	NA	<.001	<.001	<.001
HR (95% CI)	NA	NA	1 [Reference]	0.4 (0.3-0.5)	1.2 (1.0-1.4)	0.2 (0.2-0.3)
*P* value^b^	NA	NA	NA	<.001	.06	<.001
Adjusted model 1^c^						
HR (95% CI)	1 [Reference]	NA	NA	2.0 (1.8-2.4)	6.1 (5.3-7.2)	1.1 (0.9-1.3)
*P* value^b^	NA	NA	NA	<.001	<.001	.42
HR (95% CI)	NA	1 [Reference]	NA	5.4 (4.3-6.9)	16.3 (12.8-20.8)	2.9 (2.2-3.7)
*P* value^b^	NA	NA	NA	<.001	<.001	<.001
HR (95% CI)	NA	NA	1 [Reference]	0.4 (0.3-0.5)	1.2 (1.0-1.5)	0.2 (0.2-0.3)
*P* value^b^	NA	NA	NA	<.001	.03	<.001
Adjusted model 2^d^						
HR (95% CI)	1 [Reference]	NA	NA	2.8 (2.4-3.2)	6 (5.1-7.0)	1.4 (1.2-1.7)
*P* value^b^	NA	NA	NA	<.001	<.001	<.001
HR (95% CI)	NA	1 [Reference]	NA	7.5 (5.9-9.4)	16.2 (12.7-20.6)	3.8 (3.0-5.0)
*P* value^b^	NA	NA	NA	<.001	<.001	<.001
HR (95% CI)	NA	NA	1 [Reference]	0.6 (0.5-0.7)	1.2 (1.0-1.5)	0.3 (0.2-0.4)
*P* value^b^	NA	NA	NA	<.001	.04	<.001

Abbreviations: HR, hazard ratio; LDCT, low-dose computed tomography (CT) screening program; LNP, lung nodule program; Lung-RADS, Lung CT Screening Reporting and Data System.

^a^ Indicates cumulative diagnosis rate at 24 months, the typical duration of CT scan surveillance recommended for solid nodules by the Fleischner Society.^19^

^b^ Comparison with the reference value in the LDCT cohort.

^c^ Adjusted for covariables age, sex, and race and ethnicity.

^d^ Adjusted for covariables age, sex, race and ethnicity, chronic obstructive pulmonary disease, smoking status, and insurance.

**Figure 2.**
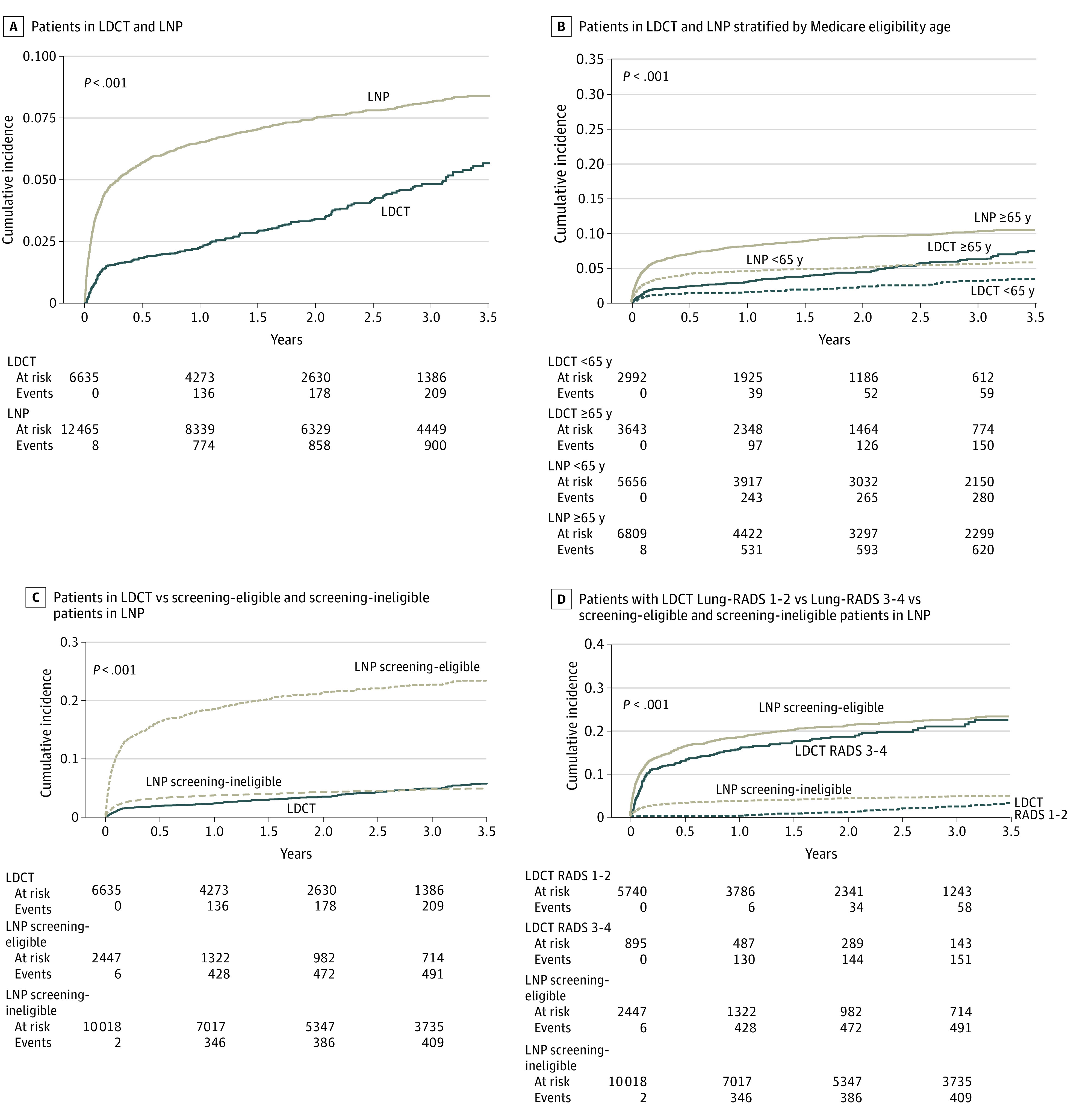
Cumulative Lung Cancer Diagnosis Rate Screening eligibility in the lung nodule program (LNP) cohort was defined according to the US Preventive Services Task Force 2021 smoking history criteria (minimum 20 pack-years, active smoking, or quit within 15 years). Persons in the LNP cohort with indeterminate smoking history were also categorized as screening ineligible. LDCT indicates low-dose computed tomography (CT) screening program; Lung-RADS, Lung CT Screening Reporting and Data System, with scores of 1 (negative), 2 (benign appearance or behavior), 3 (probably benign), and 4 (suspicious or very suspicious).

At 24 months, the cumulative incidence of lung cancer diagnosis was 3.4% (95% CI, 2.9%-3.9%) in the LDCT cohort, including 1.0% (95% CI, 0.7%-1.4%) in the Lung-RADS 1-2 and 18.6% (95% CI, 15.8%-21.5%) in the Lung-RADS 3-4 cohorts, and 7.5% (95% CI, 7.0%-8.0%) in the LNP cohort, including 21.2% (95% CI, 19.5%-22.9%) in the screening-eligible and 4.2% (95% CI, 3.8%-4.6%) in the screening-ineligible cohorts (Table 3). At all time points from 6 to 36 months, the cumulative incidence of lung cancer was higher in the whole LNP than LDCT cohorts (Figure 2A), including the Medicare eligibility age-stratified cohorts (Figure 2B). The cumulative incidence of lung cancer was highest in the screening-eligible LNP cohort, but the screening-ineligible cohort had similar cumulative lung cancer diagnosis rates as the whole LDCT cohort (Figure 2C). The cumulative diagnosis of the screening-eligible LNP cohort was greater than Lung-RADS 3-4 cohort, and the screening-ineligible LNP cohort had cumulative diagnosis rates between those of the 2 LDCT cohorts (Figure 2D).

With reference to the whole LDCT cohort, the aHR for diagnosis of lung cancer in the full model was 6.0 (95% CI, 5.1-7.0) for the screening-eligible LNP cohort and 1.4 (95% CI, 1.2-1.7) for the screening-ineligible LNP cohort. Compared with the Lung-RADS 1-2 cohort, the aHR was 16.2 (95% CI, 12.7-20.6) for the screening-eligible and 3.8 (95% CI, 3.0-5.0) for the screening-ineligible LNP cohorts. Compared with the Lung-RADS 3-4 cohort, the aHR was 1.2 (95% CI, 1.0-1.5) for the screening-eligible LNP cohort and 0.3 (95% CI, 0.2-0.4) for the screening-ineligible LNP cohort (Table 3). Compared with the Lung-RADS 3 cohort, the aHR was 1.2 (95% CI, 0.8-1.9) for the screening-ineligible LNP cohort and 4.8 (95% CI, 3.1-7.4) for the eligible LNP cohort. Compared with the LungRADS 4 cohort, the aHR was 0.7 (95% CI, 0.6-0.8) for the screening-eligible LNP cohort (eTable 2 in Supplement 1).

### Lung Cancer Characteristics

Tumor histology was adenocarcinoma in 108 of 242 patients (44.63%) in the LDCT cohort, 234 of 531 (44.07%) in the screening-eligible LNP cohort, and 234 of 447 (52.35%) in the screening-ineligible LNP cohort; findings were squamous cell carcinoma in 77 of 242 (31.82%) in the LDCT cohort, 148 of 531 (27.87%) in the screening-eligible LNP cohort, and 89 of 447 (19.91%) in the screening-ineligible LNP cohort; and findings were small cell carcinoma in 33 of 242 (13.64%) in the LDCT cohort, 72 of 531 (13.56%) in the screening-eligible LNP cohort, and 46 of 447 (10.29%) of the screening-ineligible LNP cohort (*P* = .001). Clinical stage was I or II in 156 of 242 patients (64.46%) in the LDCT cohort, 276 of 531 (52.00%) in the screening-eligible LNP cohort, and 253 of 447 (56.60%) in the screening-ineligible LNP cohort and stage IV in 40 of 242 (16.53%) in the LDCT cohort, 128 of 531 (24.11%) in the screening-eligible LNP cohort, and 109 of 447 (24.38%) in the screening-ineligible LNP cohort (*P* = .006) (eTable 3 in Supplement 1).

### Lung Cancer Diagnosis, Race and Ethnicity, and Medicare Eligibility Age

The race distribution of patients with lung cancer in the 3 cohorts differed significantly when stratified by Medicare eligibility age. The proportion of patients with lung cancer younger than 65 years vs 65 years or older in the LDCT cohort who were Black was 9 of 68 (13.24%) vs 23 of 174 (13.22%); in the screening-eligible LNP cohort, 44 of 175 (25.14%) vs 70 of 356 (19.66%); and in the screening-ineligible LNP cohort, 58 of 130 (44.62%) vs 67 of 317 (21.14%) (*P* < .001) (eTable 4 and eFigure 2 in Supplement 1). All results remained consistent when the cohort was restricted to participants without a history of cancer (eTables 5-8 in Supplement 1).

## Discussion

In this prospective cohort study, we evaluated the relative hazard of a lung cancer diagnosis among participants aged 50 to 80 years in a community health care system’s LDCT program and LNP. Compared with all participants undergoing LDCT screening, the LNP cohort was at a considerably higher hazard for lung cancer, irrespective of smoking history. Participants in the screening-eligible LNP cohort had a slightly greater hazard than those in the Lung-RADS 3-4 LDCT cohort, despite similar smoking history, family history of lung cancer, and nodule size. However, there was a slightly higher proportion of patients with COPD in the screening-eligible LNP cohort. The screening-ineligible LNP cohort had a slightly greater hazard than the Lung-RADS 1-2 LDCT cohort. In a Kaiser Permanente Southern California cohort from 2006 to 2016 with nodules greater than 8 mm, 9.9% developed lung cancer within 27 months, including 5.4% of persons who never smoked.^29^ A SEER-Medicare analysis reported a lung cancer diagnosis rate of 4.7% at 24 months.^30^ Neither study directly compared patient and nodule characteristics between LDCT and LNP, as we have done.

Compared with patients diagnosed through LDCT, significantly higher proportions of patients in the LNP cohort were Black, uninsured, or age-ineligible for enrollment into Medicare. Black persons are at higher hazard for lung cancer at a younger age and lower smoking intensity.^17,31^ The LNP provided access to early detection to large proportions of persons whose smoking history rendered them ineligible for LDCT screening: 105 of 447 (23.49%) screening-ineligible participants diagnosed with lung cancer had never smoked cigarettes, 107 (23.94%) had a history of less than 20 pack-years, and 149 (33.33%) had quit smoking for more than 15 years. These differences emphasize how an LNP expands access to early lung cancer detection. Guideline-discordant management of incidentally detected lung nodules is highly prevalent.^32,33,34,35,36^ We provide strong rationale to support institutional investment in the infrastructure required to overcome this problem, including the promotion of equitable access to early detection, a goal stated in the 2022 Medicare Coverage Decision on LDCT,^37^ the President’s Cancer Panel,^38^ and the USPSTF’s 2021 recommendations.^28^

### Limitations

This study has some limitations. We reported HRs as the mean effect size over time. Some evidence from this study suggests that the differences between LDCT and LNP could emerge in the later years of follow-up, but we did not evaluate this statistically due to the amount of data available. Guidelines currently recommend 2 years of surveillance for solid lung nodules. Missingness and uncertainty about the reliability of data, such as risk factor exposure history, are inherent in the pragmatic use of routinely generated clinical records. Missing cigarette use data in the screening-ineligible LNP cohort introduces risk for misclassification bias; an unknown proportion of these participants were probably eligible for screening. We had no information on patient-clinician interactions leading to decisions for care. We could neither quantify nor compare the rate of overdiagnosis of lung cancer in any of the cohorts.^39,40,41,42^ Furthermore, we have not formally compared survival in this analysis, focusing instead on the relative hazard of lung cancer diagnosis in the 2 program-based approaches to early lung cancer detection. However, we adjusted for the impact of competing causes of mortality, since cumulative lung cancer hazard is only possible in patients who remain alive. Finally, we may not have complete information on competing hazards for individuals who migrated out of our area. We assumed that persons not known to be dead or diagnosed with lung cancer were still alive and free of lung cancer on the censoring date.

## Conclusions

The findings of this cohort study of 2 pathways to early lung cancer detection suggest that persons enrolled in an LNP had great cumulative hazard for lung cancer diagnosis within 2 years, irrespective of smoking history. The LNP extended the possibility of early detection to LDCT-ineligible subsets whose true lung cancer hazard may have otherwise been underestimated.^24,43,44^ Such programs may provide a rich recruitment ground for screening-eligible candidates and a pathway to early lung cancer detection in populations without access to LDCT, the situation in most countries. In future studies, we plan to explore how innovative risk-stratification strategies, including artificial intelligence and biomarker-based selection, might improve the effectiveness of early lung cancer detection efforts in diverse populations.

## Notes

Supplement 1. **eAppendix.** Radiology Report Macro Text **eTable 1.** Demographic Characteristics of Enrollees Younger Than 65 Years vs 65 Years or Older in LDCT and LNP Cohorts **eTable 2.** Cumulative Incidence and Hazard of Lung Cancer Diagnosis **eTable 3.** Cancer Characteristics in LDCT and LNP Cohorts **eTable 4.** Demographic Characteristics of Patients With Lung Cancer Stratified by the Age of Medicare Eligibility in LDCT and LNP Cohorts **eTable 5.** Demographic and Clinical Characteristics of Enrollees in LDCT and LNP Cohorts, Excluding All Patients With a History of Cancer **eTable 6.** Lesion Characteristics in LDCT and LNP Cohorts, Excluding All Patients With a History of Cancer **eTable 7.** Cumulative Incidence and Hazard of Lung Cancer Diagnosis in LDCT and LNP Cohorts, Excluding All Patients With a History of Cancer **eTable 8.** Cancer Characteristics in LDCT and LNP Cohorts, Excluding All Patients With a History of Cancer **eFigure 1.** Log-Log Plot of LDCT vs LNP Cohorts **eFigure 2.** Proportion of Black and White Patients With Lung Cancer in LDCT vs LNP Cohorts, Stratified by Medicare Eligibility Age

Supplement 2.
Data Sharing Statement
