# Supplementary material for: Evaluation of Lung Cancer Risk Among Persons Undergoing Screening or Guideline-Concordant Monitoring of Lung Nodules in the Mississippi Delta
Source: JAMA Netw Open. 2023 Feb 27;6(2):e230787. doi: 10.1001/jamanetworkopen.2023.0787 (PMC9972195; doi:10.1001/jamanetworkopen.2023.0787)
Supplement: Supplement 1. — eAppendix. Radiology Report Macro Text eTable 1. Demographic Characteristics of Enrollees Younger Than 65 Years vs 65 Years or Older in LDCT and LNP Cohorts eTable 2. Cumulative Incidence and Hazard of Lung Cancer Diagnosis eTable 3. Cancer Characteristics in LDCT and LNP Cohorts eTable 4. Demographic Characteristics of Patients With Lung Cancer Stratified by the Age of Medicare Eligibility in LDCT and LNP Cohorts eTable 5. Demographic and Clinical Characteristics of Enrollees in LDCT and LNP Cohorts, Excluding All Patients With a History of Cancer eTable 6. Lesion Characteristics in LDCT and LNP Cohorts, Excluding All Patients With a History of Cancer eTable 7. Cumulative Incidence and Hazard of Lung Cancer Diagnosis in LDCT and LNP Cohorts, Excluding All Patients With a History of Cancer eTable 8. Cancer Characteristics in LDCT and LNP Cohorts, Excluding All Patients With a History of Cancer eFigure 1. Log-Log Plot of LDCT vs LNP Cohorts eFigure 2. Proportion of Black and White Patients With Lung Cancer in LDCT vs LNP Cohorts, Stratified by Medicare Eligibility Age [file jamanetwopen-e230787-s001.pdf]

Supplementary Online Content

Osarogiagbon RU, Liao W, Faris NR, et al. Evaluation of lung cancer risk among persons undergoing screening or guideline-concordant monitoring of lung nodules in the Mississippi Delta. *JAMA Netw Open*. 2023;6(2):e230787. doi:10.1001/jamanetworkopen.2023.0787

eAppendix. Radiology Report Macro Text

eTable 1. Demographic Characteristics of Enrollees Younger Than 65 Years vs 65 Years or Older in LDCT and LNP Cohorts

eTable 2. Cumulative Incidence and Hazard of Lung Cancer Diagnosis

eTable 3. Cancer Characteristics in LDCT and LNP Cohorts

eTable 4. Demographic Characteristics of Patients With Lung Cancer Stratified by the Age of Medicare Eligibility in LDCT and LNP Cohorts

eTable 5. Demographic and Clinical Characteristics of Enrollees in LDCT and LNP Cohorts, Excluding All Patients With a History of Cancer

eTable 6. Lesion Characteristics in LDCT and LNP Cohorts, Excluding All Patients With a History of Cancer

eTable 7. Cumulative Incidence and Hazard of Lung Cancer Diagnosis in LDCT and LNP Cohorts, Excluding All Patients With a History of Cancer

eTable 8. Cancer Characteristics in LDCT and LNP Cohorts, Excluding All Patients With a History of Cancer

eFigure 1. Log-Log Plot of LDCT vs LNP Cohorts

eFigure 2. Proportion of Black and White Patients With Lung Cancer in LDCT vs LNP Cohorts, Stratified by Medicare Eligibility Age

This supplementary material has been provided by the authors to give readers additional information about their work.

© 2023 Osarogiagbon RU et al. *JAMA Network Open*.

eAppendix. Radiology Report Macro Text

Certain findings detected within the thorax (chest) on this exam warrant follow-up, as per published guidelines. Additional evaluation of these findings is recommended.

© 2023 Osarogiagbon RU et al. *JAMA Network Open*.

**eTable 1.** Demographic Characteristics of Enrollees Younger Than 65 Years vs 65 Years or Older in LDCT and LNP Cohorts						
	**LDCT**		**LNP**			
	**Age < 65**	**Age >= 65**	**Screening-Eligible Age < 65**	**Screening-Eligible Age >= 65**	**Screening-Ineligible Age < 65**	**Screening-Ineligible Age >= 65**
	**N = 3009**	**N = 3675**	**N = 1122**	**N = 1375**	**N = 4590**	**N = 5558**
**Screen Age**						
Mean (SD)	59.43(3.08)	69.65(3.6)	58.01(4.17)	71.14(4.27)	57.5(4.26)	72.04(4.5)
Median (IQR)	60(57 - 62)	69(67 - 72)	58(55 - 62)	71(67 - 74)	58(54 - 61)	72(68 - 76)
(Min-Max)	(50 - 64)	(65 - 80)	(50 - 64)	(65 - 80)	(50 - 64)	(65 - 80)
**Sex (n,%)**						
Male	1448(48.12)	1927(52.44)	563(50.18)	694(50.47)	1983(43.2)	2348(42.25)
Female	1561(51.88)	1748(47.56)	559(49.82)	680(49.45)	2511(54.71)	3106(55.88)
Missing	0	0	0	1(0.07)	96(2.09)	104(1.87)
**Race (n, %)**						
Black	578(19.21)	666(18.12)	235(20.94)	257(18.69)	1585(34.53)	1329(23.91)
White	2387(79.33)	2950(80.27)	866(77.18)	1099(79.93)	2714(59.13)	3959(71.23)
Other/not reported	44(1.46)	59(1.60)	21(1.87)	19(1.38)	291(6.34)	270(4.86)
**Ethnicity (n, %)**						
Hispanic/Latino	14(0.47)	14(0.38)	9(0.8)	4(0.29)	78(1.7)	54(0.97)
Not Hispanic/Latino	2939(97.67)	3586(97.58)	1101(98.13)	1366(99.35)	4355(94.88)	5342(96.11)
Not reported	56(1.86)	75(2.14)	12(1.07)	5(0.36)	157(3.42)	162(2.92)
**Insurance (n, %)**						
Medicare	913(30.34)	3155(85.85)	338(30.12)	1211(88.07)	792(17.25)	4562(82.08)
Medicaid	186(6.18)	14(0.38)	86(7.66)	2(0.15)	164(3.57)	19(0.34)
Commercial	1832(60.88)	492(13.39)	598(53.3)	148(10.76)	2987(65.08)	725(13.04)
Uninsured	78(2.59)	14(0.38)	100(8.91)	14(1.02)	647(14.1)	252(4.53)

© 2023 Osarogiagbon RU et al. *JAMA Network Open*.

eTable 2. Cumulative Incidence and Hazard of Lung Cancer Diagnosis

	LDCT					LNP		
	**Total**	**RADS 1**	**RADS 2**	**RADS 3**	**RADS 4**	**Total**	**Screening-Eligible**	**Screening-Ineligible**
	**N = 6684**	**N = 2582**	**N = 3192**	**N = 455**	**N = 455**	**N = 12645**	**N = 2497**	**N = 10148**
**Duration of follow-up from Enrollment to Diagnosis, days**								
Mean (SD)	396.53(451.44)	941.31(478)	726(337.29)	535.29(395.08)	138.44(249.04)	195.88(338.99)	197.19(360.49)	194.28(311.02)
Median (IQR)	188.5(37- 658)	823.5(642 - 1208.25)	685(444.5 - 955.5)	401(317 - 675)	42(20 - 114)	45(14 - 199)	41(12 - 182)	50.5(16 - 208.25)
(Min - Max)	(4 - 2131)	(142 - 2131)	(35 - 1490)	(36 - 1793)	(4 - 1415)	(0 - 2507)	(0 - 2507)	(0 - 1539)
**Cumulative Incidence of Lung Cancer Diagnosis (95%CI)**								
1 6 months	1.8(1.5, 2.2)	0.04(0.0, 0.25)	0.07(0.02, 0.25)	0.5(0.1, 1.7)	26.0(22.0, 30.0)	5.7(5.3, 6.1)	16.0(15.0, 18.0)	3.1(2.8, 3.5)
2 12 months	2.2(1.9, 2.6)	0.2(0.0, 0.4)	0.1(0.0, 0.3)	2.3(1.1, 4.4)	29.2(24.8, 33.7)	6.5(6.1, 7.0)	18.5(16.9, 20.1)	3.6(3.3, 4.0)
3 18 months	2.9(2.5, 3.4)	0.3(0.14, 0.71)	0.9(0.52, 1.4)	4.6(2.6, 7.4)	30.0(26.0, 35.0)	7.0(6.6, 7.5)	20.0(19.0, 22.0)	3.9(3.5, 4.3)
4 24 months	3.4(2.9, 3.9)	0.7(0.4, 1.3)	1.3(0.8, 1.9)	5.5(3.2, 8.6)	31.7(27.1, 36.5)	7.5(7.0, 8.0)	21.2(19.5, 22.9)	4.2(3.8, 4.6)
5 30 months	4.2(3.6, 4.9)	1.5(0.91, 2.3)	2.1(1.4, 2.9)	6.6(4.0, 10.0)	33.0(28.0, 38.0)	7.8(7.3, 8.3)	22.0(20.0, 24.0)	4.4(4.0, 4.9)
6 36 months	4.8(4.1, 5.6)	1.7(1.1, 2.6)	2.8(2.0, 3.9)	7.4(4.5, 11.4)	34.6(29.3, 39.9)	8.2(7.6, 8.7)	22.7(20.9, 24.5)	4.7(4.2, 5.1)
**Unadjusted Cox Model**								
HR (95% CI) p-value								
	Ref					2(1.77,2.36)	6.3(5.36,7.32)	1.1(0.93,1.28)
						<0.001	<0.001	0.28
		Ref				6(4.2,8.5)	18.3(12.82,26.2)	3.2(2.26,4.65)
						<0.001	<0.001	<0.001
			Ref			5.2(3.82,7)	15.8(11.63,21.54)	2.8(2.04,3.81)

© 2023 Osarogiagbon RU et al. *JAMA Network Open*.

						<0.001	<0.001	<0.001
				Ref		1.5(1,2.39)	4.8(3.07,7.35)	0.8(0.54,1.31)
						0.05	<0.001	0.45
					Ref	0.2(0.17,0.25)	0.6(0.52,0.76)	0.1(0.09,0.14)
						<0.001	<0.001	<0.001
**Adjusted Cox Model 1: Covariables: age, sex, race**								
aHR (95% CI) p-value								
	Ref					2(1.76,2.36)	6.1(5.26,7.19)	1.1(0.91,1.26)
						<0.001	<0.001	ge
		Ref				5.9(4.13,8.36)	17.7(12.38,25.31)	3.1(2.17,4.47)
						<0.001	<0.001	<0.001
			Ref			5.2(3.85,7.06)	15.7(11.5,21.3)	2.8(2.01,3.77)
						<0.001	<0.001	<0.001
				Ref		1.6(1.02,2.43)	4.8(3.09,7.4)	0.8(0.55,1.32)
						0.04	<0.001	0.46
					Ref	0.2(0.18,0.26)	0.7(0.55,0.81)	0.1(0.1,0.14)
						<0.001	<0.001	<0.001
**Adjusted Cox Model 2: Covariables: age, sex, race, COPD, smoking status, insurance**								
aHR (95% CI) p-value								
	Ref					2.8(2.4,3.22)	6(5.14,7.04)	1.4(1.19,1.7)
						<0.001	<0.001	<0.001
		Ref				8.1(5.72,11.62)	17.5(12.24,25.06)	4.2(2.9,6.09)

© 2023 Osarogiagbon RU et al. *JAMA Network Open*.

						<0.001	<0.001	<0.001
			Ref			7.1(5.21,9.58)	15.5(11.4,21.17)	3.7(2.64,5.04)
						<0.001	<0.001	<0.001
				Ref		2.3(1.48,3.53)	4.8(3.07,7.36)	1.2(0.76,1.86)
						<0.001	<0.001	0.45
					Ref	0.3(0.26,0.38)	0.7(0.55,0.8)	0.2(0.13,0.19)
						<0.001	<0.001	<0.001

Yellow highlight indicates cumulative diagnosis rate at 24 months, the typical duration of CT scan surveillance recommended for solid nodules by the Fleischner Society.[19] aHR = adjusted hazard ratio; COPD = Chronic Obstructive Pulmonary Disease; HR= hazard ratio; LDCT= low-dose CT screening program; LNP = lung nodule program; Lung-RADS= Lung CT Screening Reporting and Data System; Ref= reference cohort.

© 2023 Osarogiagbon RU et al. *JAMA Network Open*.

**eTable 3.** Cancer Characteristics in LDCT and LNP Cohorts			
	**LDCT**	**LNP**	
	**N = 242**	**Screening-Eligible**	**Screening-Ineligible**
		**N = 531**	**N = 447**
**Histology (n, %)**			
Adenocarcinoma	108(44.63)	234(44.07)	234(52.35)
Squamous	77(31.82)	148(27.87)	89(19.91)
Adenosquamous	0	4(0.75)	5(1.12)
Large cell	7(2.89)	17(3.2)	9(2.01)
Small cell	33(13.64)	72(13.56)	46(10.29)
Other	16(6.61)	54(10.17)	63(14.09)
Not Reported	1(0.41)	2(0.38)	1(0.22)
**Primary Tumor Size (cm)**			
Mean (SD)	2.37(1.61)	3.43(2.71)	3.34(2.44)
Median (IQR)	1.9(1.3 - 2.9)	2.5(1.6 - 4.5)	2.5(1.6 - 4.25)
Missing	12(4.96)	21(3.95)	24(5.37)
**Clinical T Category (n, %)**			
cTX/0/is	2(0.83)	8(1.51)	15(3.36)
cT1	163(67.36)	280(52.73)	230(51.45)
cT2b	30(12.4)	83(15.63)	81(18.12)
cT3	20(8.26)	64(12.05)	43(9.62)
cT4	22(9.09)	91(17.14)	78(17.45)
Not Reported	5(2.07)	5(0.94)	0
**Clinical N Category (n, %)**			
cN0	166(68.6)	328(61.77)	312(69.8)
cN1	14(5.79)	33(6.21)	16(3.58)
cN2	31(12.81)	104(19.59)	68(15.21)
cN3	26(10.74)	61(11.49)	51(11.41)
Not Reported	5(2.07)	5(0.94)	0
**Clinical M Category (n, %)**			
cM0	196(80.99)	389(73.26)	335(73.63)
cM1a	8(3.31)	29(5.46)	29(6.37)
cM1b	8(3.31)	39(7.34)	26(5.71)
cM1c	25(10.33)	69(12.99)	65(14.29)
Not Reported	5(2.07)	5(0.94)	0
**Clinical Stage (n, %)**			
Occult/Stage 0	0	0	1(0.22)
Stage I	137(56.61)	236(44.44)	220(49.22)
Stage II	19(7.85)	40(7.53)	33(7.38)
Stage III	40(16.53)	122(22.98)	82(18.34)
Stage IV	40(16.53)	128(24.11)	109(24.38)

© 2023 Osarogiagbon RU et al. *JAMA Network Open*.

Not Reported	6(2.48)	5(0.94)	2(0.45)
LDCT = low-dose CT screening program; LNP = lung nodule program.			

© 2023 Osarogiagbon RU et al. *JAMA Network Open*.

**eTable 4.** Demographic Characteristics of Patients With Lung Cancer Stratified by the Age of Medicare Eligibility in LDCT and LNP Cohorts						
	**LDCT**		**LNP**			
	**Age < 65**	**Age >= 65**	**Screening-Eligible Age < 65**	**Screening-Eligible Age >= 65**	**Screening-Ineligible Age < 65**	**Screening-Ineligible Age >= 65**
	**N = 68**	**N = 174**	**N = 175**	**N = 356**	**N = 130**	**N = 317**
**Screen Age**						
Mean (SD)	60.19(2.86)	70.7(3.41)	59.14(3.77)	71.35(4.28)	58.89(4.06)	72.84(4.57)
Median (IQR)	61(58 - 63)	70.5(68 - 73)	60(56 - 62)	71(68 - 75)	59(56 - 62)	73(69 - 77)
(Min-Max)	(55 - 64)	(65 - 79)	(50 - 64)	(65 - 80)	(50 - 64)	(65 - 80)
**Sex (n,%)**						
Male	34(50)	93(53.45)	84(48)	176(49.44)	51(39.23)	157(49.53)
Female	34(50)	81(46.55)	91(52)	179(50.28)	79(60.77)	160(50.47)
Missing	0	0	0	1(0.28)	0	0
**Race (n, %)**						
Black	9(13.24)	23(13.22)	44(25.14)	70(19.72)	58(44.62)	67(21.14)
White	59(86.76)	151(86.78)	128(73.14)	284(79.78)	65(50)	246(77.6)
Other/NR	0	0	3(1.71)	2(0.56)	7(5.39)	4(1.27)
**Ethnicity (n, %)**						
Hispanic/Latino	0	0	2(1.14)	1(0.28)	2(1.54)	2(0.63)
Not Hispanic/Latino	72(100)	172(98.85)	171(97.71)	353(99.44)	127(97.69)	314(99.05)
Other/NR	0	2(1.15)	2(1.14)	1(0.28)	1(0.77)	1(0.32)
**Insurance (n, %)**						
Medicare	15(22.06)	154(88.51)	54(30.86)	322(90.7)	28(21.54)	272(85.8)
Medicaid	9(13.24)	0	14(8)	1(0.28)	6(4.62)	2(0.63)
Commercial	44(64.71)	20(11.49)	89(50.86)	30(8.45)	86(66.15)	39(12.3)
Uninsured	0	0	18(10.29)	2(0.56)	10(7.69)	4(1.26)

© 2023 Osarogiagbon RU et al. *JAMA Network Open*.

**eTable 5.** Demographic and Clinical Characteristics of Enrollees in LDCT and LNP Cohorts, Excluding All Patients With a History of Cancer			
	**LDCT[a]**	**LNP[a]**	
	**N = 5451**	**Screening-Eligible**	**Screening-Ineligible**
		**N = 1927**	**N = 8057**
**Age**			
Mean (SD)	64.66(6.04)	64.59(7.76)	64.76(8.45)
Median (IQR)	65(60 - 69)	65(58 - 71)	65(58 - 72)
(Min-Max)	(50 - 80)	(50 - 80)	(50 - 80)
**Sex**			
Male	2768(50.78)	982(50.96)	3407(42.29)
Female	2683(49.22)	944(48.99)	4450(55.23)
Missing	0	1(0.05)	200(2.48)
**Race**			
Black	1074(19.7)	388(20.13)	2382(29.56)
White	4281(78.54)	1508(78.26)	5157(64.01)
Other[b]/Not Reported	96(1.76)	31(1.60)	518(6.44)
**Ethnicity**			
Hispanic/Latino	26(0.48)	12(0.62)	114(1.41)
Not Hispanic/Latino	5313(97.47)	1900(98.6)	7643(94.86)
Not Reported	112(2.06)	15(0.77)	300(3.72)
**Insurance**			
Medicare	3213(58.94)	1144(59.37)	4009(49.76)
Medicaid	174(3.19)	78(4.05)	152(1.89)
Commercial	1984(36.4)	609(31.6)	3060(37.98)
Uninsured	80(1.47)	96(4.98)	836(10.38)
**Smoking Status**			
Active	3661(67.16)	1396(72.44)	1498(18.59)
Former	1754(32.18)	531(27.56)	2183(27.09)
Never	20(0.37)	0	3651(45.31)
Unknown	16(0.29)	0	725(9)
**Pack years [c]**	**N[c] = 5415**	**N[c] = 1927**	**N[c] = 3681**
0 - 9	42(0.82)	0	484(13.14)
10 - 19	110(2.03)	0	432(11.74)
20 - 29	209(3.86)	337(17.49)	138(3.75)
30 - 40	1085(20.04)	321(16.66)	110(2.99)
> 40	3374(62.31)	1269(65.85)	206(5.6)
Missing	593(10.95)	0	2311(62.78)
**Quit Duration [d]**	**N[d] = 1754**	**N[d] = 531**	**N[d] = 2183**
0 - 15 years	1555(88.65)	531(100)	361(16.54)

© 2023 Osarogiagbon RU et al. *JAMA Network Open*.

16 -25 years	103(5.87)	0	349(15.99)
> 25 years	56(3.19)	0	613(28.08)
Missing	40(2.28)	0	860(39.4)
**Exposure type**			
Second-hand Smoke	203(3.72)	115(5.97)	1404(17.43)
Asbestos Exposure	58(1.06)	28(1.45)	45(0.56)
Radon Exposure	1(0.02)	0	0
Uranium Exposure	0	0	0
**Comorbidity Distribution**			
Mean (SD)	1.13(1.17)	1.57(1.32)	0.92(1.16)
Median (IQR)	1(0 - 2)	1(1 - 2)	1(0 - 1)
(Min-Max)	(0 - 7)	(0 - 8)	(0 - 8)
**Chronic Obstructive Pulmonary Disease**			
No	3185(58.43)	913(47.38)	6926(85.96)
Yes	2266(41.57)	1014(52.62)	1131(14.04)
**Charlson Comorbidity Score**			
0	1916(35.15)	403(20.91)	3868(48.01)
1	2727(50.03)	1050(54.49)	3127(38.81)
2	808(14.82)	474(24.6)	1062(13.18)
**Prior history of cancer**			
No	5451(100)	1927(100)	8057(100)
Yes	0	0	0
**Family history of cancer**			
No	1460(26.78)	589(30.57)	2616(32.47)
Yes	2494(45.75)	971(50.39)	2609(32.38)
Unknown	1497(27.46)	367(19.05)	2832(35.15)
**Family lung cancer**			
No	1862(34.16)	721(37.42)	2106(26.14)
Yes	632(11.59)	250(12.97)	503(6.24)

[a] Number (%), unless otherwise stated; [b]Other includes Asian, Native Hawaiian; [c]denominator is persons who smoke(d); [d]denominator is persons who quit smoking. LDCT = low-dose CT screening program; LNP = lung nodule program.

© 2023 Osarogiagbon RU et al. *JAMA Network Open*.

**eTable 6.** Lesion Characteristics in LDCT and LNP Cohorts, Excluding All Patients With a History of Cancer						
	**LDCT[a]**			**LNPa**		
	**Total**	**RADS 1-2**	**RADS 3-4**	**Total**	**Screening-Eligible**	**Screening-Ineligible**
	**N = 5451**	**N = 4716**	**N = 735**	**N = 9984**	**N = 1927**	**N = 8057**
**Lung RADS category**						
1 Normal, annual screening	2119(38.87)	2119(44.93)	0			
2 Benign, annual screening	2597(47.64)	2597(55.07)	0			
3 6 mons CT	379(6.95)	0	379(51.56)			
4 4A/B/X 3 months CT ~ Diagnostic test	356(6.53)	0	356(48.44)			
**Number (n, %)[b]**						
0/not reported	2676(49.09)	2643(56.04)	33(4.49)	1355(13.57)	204(10.59)	1151(14.29)
1	1140(20.91)	855(18.13)	285(38.78)	5842(58.51)	1132(58.74)	4710(58.46)
>1	1635(29.99)	1218(25.83)	417(56.73)	2787(27.91)	591(30.67)	2196(27.26)
**Size of largest[b] (mm)**	**N[a] = 2775**	**N[a] = 2073**	**N[a] = 702**	**N[a] = 8629**	**N[a] = 1723**	**N[a] = 6906**
Mean (SD)	6.31(15.45)	3.81(3.61)	13.79(28.95)	11.01(12.51)	14.62(16.3)	10.1(11.18)
Median (IQR)	4(2 - 6)	3(2 - 4)	9(6 - 15)	7(5 - 12)	9(6 - 16)	7(5 - 11)
Missing	59(2.13)	39(1.88)	20(2.85)	662(7.67)	128(7.43)	534(7.73)
**Location [b]**	**N[a] = 2775**	**N[a] = 2073**	**N[a] = 702**	**N[a] = 8629**	**N[a] = 1723**	**N[a] = 6906**
Right upper lobe	902(32.5)	697(33.62)	205(29.2)	2026(23.48)	500(29.02)	1526(22.1)
Right middle lobe	282(10.16)	227(10.95)	55(7.83)	1024(11.87)	141(8.18)	883(12.79)
Right lower lobe	510(18.38)	365(17.61)	145(20.66)	2044(23.69)	347(20.14)	1697(24.57)
Left upper lobe	601(21.66)	449(21.66)	152(21.65)	1490(17.27)	401(23.27)	1089(15.77)
Left lower lobe	434(15.64)	302(14.57)	132(18.8)	1657(19.2)	257(14.92)	1400(20.27)
Mediastinum	13(0.47)	6(0.29)	7(1)	135(1.56)	30(1.74)	105(1.52)
Not reported	33(1.19)	27(1.3)	6(0.85)	253(2.94)	47(2.73)	206(2.99)

[a] Number (%), unless otherwise stated; [b] among persons with at least 1 reported nodule. LDCT = low-dose CT screening program; LNP = lung nodule program; Lung-RADS = Lung CT Screening Reporting and Data System.

© 2023 Osarogiagbon RU et al. *JAMA Network Open*.

**eTable 7**. Cumulative Incidence and Hazard of Lung Cancer Diagnosis in LDCT and LNP Cohorts, Excluding All Patients With a History of Cancer						
	**LDCT**			**LNP**		
	**Total**	**RADS 1-2**	**RADS 3-4**	**Total**	**Screening-Eligible**	**Screening-Ineligible**
	**N = 5451**	**N = 4716**	**N = 735**	**N = 9984**	**N = 1927**	**N = 8057**
**Duration of follow-up from Enrollment to Diagnosis, days**						
Mean (SD)	382.18(439.65)	818.18(394.03)	202.85(314.77)	186.32(332.53)	186.04(347.04)	186.67(314.67)
Median (IQR)	170(35.5 - 628)	779(474.5 - 1086)	50(23.75 - 245.75)	40(12.75 - 174.5)	37(12 - 170.25)	43.5(14 - 179.75)
(Min - Max)	(4 - 1981)	(142 - 1981)	(4 - 1793)	(0 - 1978)	(0 - 1978)	(0 - 1539)
**Cumulative Incidence of Lung Cancer Diagnosis (95%CI)**						
1 6 months	1.7(1.4,2.1)	0.02(0.0, 0.14)	13.0(10.0, 15.0)	5.8(5.3, 6.3)	17.0(15.0, 18.0)	3.2(2.8, 3.6)
2 12 months	2.1(1.7, 2.5)	0.1(0.0, 0.2)	15.0(12.4, 17.9)	6.5(6.0, 7.0)	18.7(16.9, 20.5)	3.6(3.2, 4.1)
3 18 months	2.7(2.3, 3.3)	0.53(0.32, 0.85)	17.0(14.0, 19.0)	6.9(6.4, 7.5)	20.0(18.0, 22.0)	3.8(3.4, 4.3)
4 24 months	3.2(2.7, 3.7)	0.8(0.5, 1.2)	18.6(15.5, 21.9)	7.4(6.8, 7.9)	21.1(19.2, 23.1)	4.2(3.7, 4.6)
5 30 months	4.0(3.3, 4.7)	1.5(1.1, 2.1)	20.0(17.0, 24.0)	7.7(7.1, 8.2)	22.0(20.0, 24.0)	4.3(3.9, 4.8)
6 36 months	4.4(3.7, 5.2)	1.8(1.3, 2.5)	21.1(17.5, 24.9)	8.0(7.4, 8.6)	22.3(20.3, 24.4)	4.6(4.1, 5.1)
**Unadjusted Cox Model**						
HR (95% CI) p-value						
	Ref			2.2(1.84,2.57)	6.7(5.63,8.05)	1.2(0.97,1.41)
				<0.001	<0.001	0.09
		Ref		6.6(4.93,8.7)	20.2(15.1,27.08)	3.5(2.62,4.74)
				<0.001	<0.001	<0.001
			Ref	0.4(0.32,0.47)	1.2(0.98,1.46)	0.2(0.17,0.26)
				<0.001	0.08	<0.001
**Adjusted Cox Model 1: Covariables: age, sex, race**						
aHR (95% CI) p-value						
	Ref			2.2(1.86,2.6)	6.7(5.58,7.97)	1.2(0.96,1.4)
				<0.001	<0.001	0.12
		Ref		6.6(4.98,8.8)	19.9(14.89,26.72)	3.5(2.59,4.7)
				<0.001	<0.001	<0.001

© 2023 Osarogiagbon RU et al. *JAMA Network Open*.

			Ref	0.4(0.34,0.49)	1.2(1.02,1.52)	0.2(0.18,0.27)
				<0.001	0.04	<0.001
**Adjusted Cox Model 2: Covariables: age, sex, race, COPD, smoking status, insurance**						
aHR (95% CI) p-value						
	Ref			2.9(2.46,3.45)	6.5(5.45,7.82)	1.5(1.22,1.83)
				<0.001	<0.001	<0.001
		Ref		8.8(6.59,11.7)	19.8(14.75,26.51)	4.5(3.31,6.15)
				<0.001	<0.001	<0.001
			Ref	0.6(0.47,0.69)	1.2(1.01,1.52)	0.3(0.23,0.36)
				<0.001	0.04	<0.001

Yellow highlight indicates cumulative diagnosis rate at 24 months, the typical duration of CT scan surveillance recommended for solid nodules by the Fleischner Society.[19] aHR = adjusted hazard ratio; COPD = Chronic Obstructive Pulmonary Disease; HR= hazard ratio; LDCT= low-dose CT screening program; LNP = lung nodule program; Lung-RADS= Lung CT Screening Reporting and Data System; Ref= reference cohort.

© 2023 Osarogiagbon RU et al. *JAMA Network Open*.

**eTable 8.** Cancer Characteristics in LDCT and LNP Cohorts, Excluding All Patients With a History of Cancer			
	**LDCT**	**LNP**	
	**N = 183**	**Screening-Eligible**	**Screening-Ineligible**
		**N = 409**	**N = 353**
**Histology (n, %)**			
Adenocarcinoma	87(47.54)	180(44.01)	187(52.97)
Squamous	52(28.42)	118(28.85)	66(18.7)
Adenosquamous	0	3(0.73)	2(0.57)
Large cell	6(3.28)	11(2.69)	6(1.7)
Small cell	24(13.11)	54(13.2)	39(11.05)
Other	14(7.65)	41(10.02)	52(14.73)
Not Reported	0	2(0.49)	1(0.28)
**Primary Tumor Size (cm)**			
Mean (SD)	2.45(1.69)	3.57(2.81)	3.43(2.53)
Median (IQR)	2(1.3 - 2.9)	2.75(1.6 - 4.8)	2.55(1.67 - 4.4)
Missing	10(5.46)	19(4.65)	21(5.95)
**Clinical T Category (n, %)**			
cTX/0/is	1(0.55)	7(1.71)	12(3.4)
cT1	123(67.21)	204(49.88)	180(50.99)
cT2b	22(12.02)	65(15.89)	64(18.13)
cT3	13(7.1)	54(13.2)	33(9.35)
cT4	19(10.38)	76(18.58)	64(18.13)
Not Reported	5(2.73)	3(0.73)	0
**Clinical N Category (n, %)**			
cN0	127(69.4)	250(61.12)	236(66.86)
cN1	9(4.92)	26(6.36)	14(3.97)
cN2	25(13.66)	81(19.8)	56(15.86)
cN3	17(9.29)	49(11.98)	47(13.31)
Not Reported	5(2.73)	3(0.73)	0
**Clinical M Category (n, %)**			
cM0	149(81.42)	299(73.11)	255(72.24)
cM1a	5(2.73)	22(5.38)	25(7.08)
cM1b	4(2.19)	30(7.33)	22(6.23)
cM1c	20(10.93)	55(13.45)	51(14.45)
Not Reported	5(2.73)	3(0.73)	0
**Clinical Stage (n, %)**			
Occult/Stage 0	0	0	1(0.28)
Stage I	105(57.38)	175(42.79)	170(48.16)
Stage II	14(7.65)	35(8.56)	25(7.08)
Stage III	30(16.39)	98(23.96)	67(18.98)
Stage IV	29(15.85)	98(23.96)	88(24.93)

© 2023 Osarogiagbon RU et al. *JAMA Network Open*.

Not Reported	5(2.73)	3(0.73)	2(0.57)
LDCT = low-dose CT screening program; LNP = lung nodule program.			

© 2023 Osarogiagbon RU et al. *JAMA Network Open*.

eFigure 1. Log-Log Plot of LDCT vs LNP Cohorts

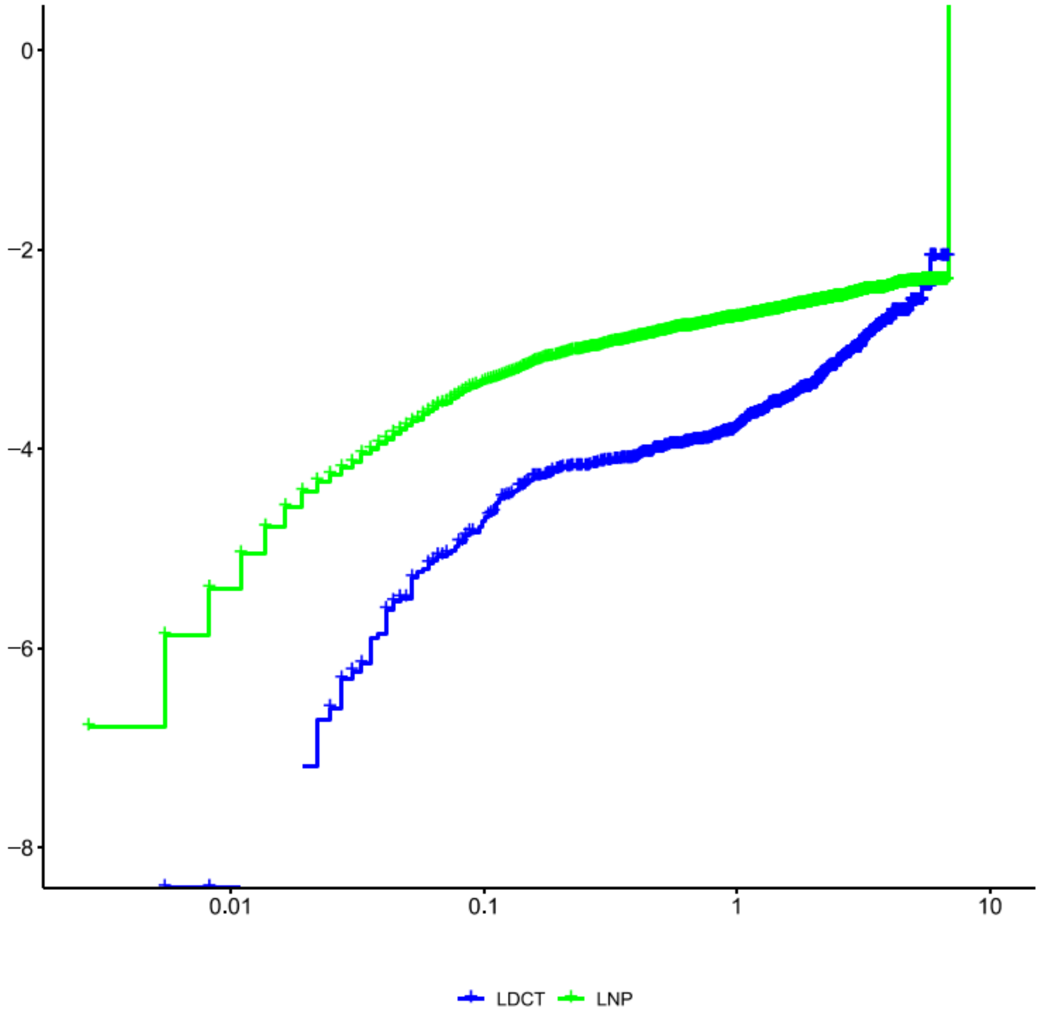

© 2023 Osarogiagbon RU et al. *JAMA Network Open*.

eFigure 2. Proportion of Black and White Patients With Lung Cancer in LDCT vs LNP Cohorts, Stratified by Medicare Eligibility Age

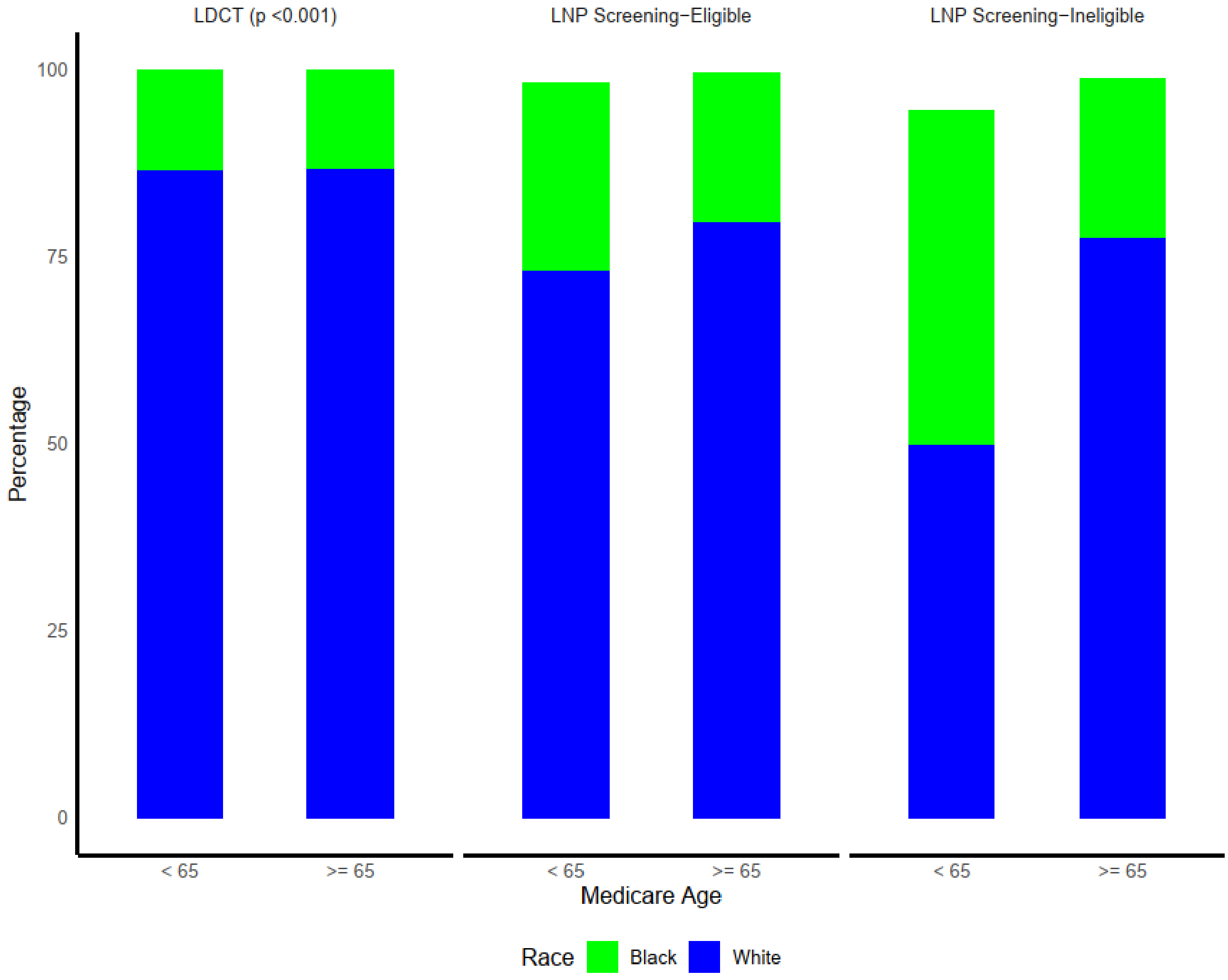

© 2023 Osarogiagbon RU et al. *JAMA Network Open*.
