# Supplementary material for: Evaluation of Lung Cancer Risk Among Persons Undergoing Screening or Guideline-Concordant Monitoring of Lung Nodules in the Mississippi Delta
Source: JAMA Netw Open. 2023 Feb 27;6(2):e230787. doi: 10.1001/jamanetworkopen.2023.0787 (PMC9972195; doi:10.1001/jamanetworkopen.2023.0787)
Supplement: Supplement 2. — Data Sharing Statement [file jamanetwopen-e230787-s002.pdf]

Data Sharing Statement

Osarogiagbon. Evaluation of Lung Cancer Risk Among Persons Undergoing Screening or Guideline-Concordant Monitoring of Lung Nodules in the Mississippi Delta. *JAMA Netw Open*. Published February 27, 2023. doi:10.1001/jamanetworkopen.2023.0787

Data
Data available: No
